# The Challenges of Algorithm-Based HR Decision-Making for Personal Integrity

**DOI:** 10.1007/s10551-019-04204-w

**Published:** 2019-06-07

**Authors:** Ulrich Leicht-Deobald, Thorsten Busch, Christoph Schank, Antoinette Weibel, Simon Schafheitle, Isabelle Wildhaber, Gabriel Kasper

**Affiliations:** 1 INSEAD, Fontainebleau, France; 2 IWE-HSG, University of St. Gallen, St. Gallen, Switzerland; 3 University of Vechta, Vechta, Germany; 4 FAA-HSG, University of St. Gallen, St. Gallen, Switzerland

**Keywords:** Algorithm-based decision-making, Personal integrity, Moral imagination, Critical algorithm studies, Workplace monitoring

## Abstract

Organizations increasingly rely on algorithm-based HR decision-making to monitor their employees. This trend is reinforced by the technology industry claiming that its decision-making tools are efficient and objective, downplaying their potential biases. In our manuscript, we identify an important challenge arising from the efficiency-driven logic of algorithm-based HR decision-making, namely that it may shift the delicate balance between employees’ personal integrity and compliance more in the direction of compliance. We suggest that critical data literacy, ethical awareness, the use of participatory design methods, and private regulatory regimes within civil society can help overcome these challenges. Our paper contributes to literature on workplace monitoring, critical data studies, personal integrity, and literature at the intersection between HR management and corporate responsibility.

Data have been discussed as “the new oil” (Tarnoff 2017; Thorp 2012) that organizations need to extract and monetize using algorithms or sets of defined steps structured to process data (Gillespie 2014). As a result, modern workplaces increasingly become quantified and monitored by algorithms (Ball 2010). For example, the technology firm Xerox Services applied a recruitment algorithm to support HR managers in their hiring decisions, offering them a score of how well an applicant’s qualifications fit to a job (Peck 2013). Moreover, the bank JP Morgan applies a fraud prediction algorithm to identify whether its employees behave in accordance with the company’s compliance regulations (Son 2015). Against this background, scholars in the fields of business ethics (Martin and Freeman 2003), critical algorithm studies (Ananny 2016; Kitchin 2017; Willson 2017), workplace monitoring (Ball 2001), and management (Bernstein 2017) have discussed the use of algorithm-based decision-making, problematizing issues regarding privacy (Martin and Nissenbaum 2016), accountability (Diakopoulos 2016; Neyland 2015), transparency (Ananny and Crawford 2018; Martin 2018; Stohl et al. 2016), power (Beer 2017; Neyland and Möllers 2017), and social control (Ajunwa et al. 2017; boyd and Crawford 2012; Zuboff 1988).

Technology firms and business consultants have, by contrast, predominantly painted a “rosy and often naively optimistic and ultimately rationalistic picture of the business role and functions of big data” (Constantiou and Kallinikos 2015, p. 53), praising the technological sophistication and usefulness of algorithm-based decision-making. The technology firm IBM (2018), for example, advertises its HR artificial intelligence algorithm Talent Watson as empowering “HR teams to increase the efficiency and quality of their operations.” In a similar vein, the analytics provider SAS (2018) claims that “fact-based decisions, powered by analytics, enable organizations to more accurately define their strategy and be successful.” Novel technological advancements, however, do not simply offer opportunities for more effective organizing but also come with broader social and cultural implications (Dourish 2016; Martin and Freeman 2004; Orlikowski 2007; Verbeek 2006). Zuboff (2015) reminds us that implementing a novel technology is not an autonomous process that humans have no control over. Instead, such an implementation is also a social process that organizational members can actively participate in, object to, and game with (Friedman et al. 2013; Shilton and Anderson 2017).

In this paper, we analyze how algorithm-based HR decision-making (i.e., algorithms designed to support and govern HR decisions), may influence employees’ personal integrity, defined as a person’s consistency between convictions, words, and actions (Palanski and Yammarino 2009). As Margolis et al. (2007, p. 237) put it, HR management has “the potential to change, shape, redirect and fundamentally alter the course of other people’s lives.” Hence, we expect that algorithm-based HR decision-making has profound effects on those governed by these decisions: the employees. We focus on personal integrity as an outcome because it is an innate human ability to make sense of one’s own decisions, behavior, and actions. According to Koehn (2005), personal integrity is a necessity for truly being human. Following this view, we suggest that although personal integrity may be useful for organizations, above all it is a fundamental human value for its own sake.

We claim that algorithm-based HR decision-making can shift the delicate balance between employees’ personal integrity and compliance more toward the compliance side because it may evoke blind trust in processes and rules, which may ultimately marginalize human sense-making as part of the decision-making processes. This is particularly true because algorithms lack the capacity for moral imagination (i.e., to be aware of contextual moral dilemmas and to create new solutions). Thus, HR managers’ reliance on algorithm-based decision-making may crowd-out employees’ personal integrity in favor of compliance, which is limited to employees’ conforming to externally generated rules and regulation.

Our manuscript offers three important theoretical contributions. First, our paper extends prior workplace monitoring and critical algorithm literature by showing how current algorithm-based HR decision-making applications can limit employees’ personal integrity. This is vitally important as the line between monitoring employees at the workplace and in private has increasingly become blurred (Rosenblat et al. 2014). As such, employees cannot easily opt out of workplace monitoring, if at all (Ajunwa et al. 2017). Thus, harming personal integrity at work might also have significant spill-over effects on employees’ private lives (Rosenblat and Stark 2016). Furthermore, critical algorithm studies have examined algorithms directed toward constituents outside the organization, such as platform users (Bucher 2012, 2017; Mager 2012; Willson 2017), customers (Crawford 2015), consumers (Carah 2015), or freelance workers (Kushner 2013) but less on algorithms influencing employees and managers *within* organizations. Our manuscript joins prior business ethicists’ assessments (Leclercq-Vandelannoitte 2017; Martin and Freeman 2003; Ottensmeyer and Heroux 1991) suggesting that algorithm-based HR decision-making is conducive to social control, creating what Zuboff (1988, p. 323) refers to as “anticipatory conformity.”

Second, our manuscript contributes to the literature on integrity and compliance by exploring the consequences of algorithm-based HR decision-making for personal integrity. We suggest that the novel challenges of algorithm-based HR decision-making for personal integrity go beyond factors that have already been described in literature, factors such as rigid organizational structures or employees’ own self-interested behavior (Adler and Borys 1996). Even before the advent of big data, institutional structures of HR practices have partly compromised employees’ personal integrity (Wilcox 2012). However, we suggest that while algorithm-based HR decision-making aggravates some of the already known quandaries (Ekbia et al. 2015), it also creates novel tensions, such as increased information asymmetries between management and employees, thereby reducing employees’ sense of autonomy and, hence, further shifting the delicate balance between integrity and compliance toward compliance.

Finally, our paper contributes to literature at the intersection between HR management and corporate responsibility by highlighting employees’ personal integrity as a central intrinsic value to enact moral agency. Greenwood (2002) suggested that HR management tends to implicitly draw from normative assumptions of consequentialist and deontological ethics, highlighting criteria of efficiency and fairness when assessing HR-related processes, such as employee recruitment, evaluation or performance appraisals (Legge 1996; Miller 1996). Instead, our analysis is loosely rooted in discourse ethics (Beschorner 2006; Busch and Shepherd 2014; Scherer 2015) suggesting that personal integrity is a human potentiality in its own right that should be bolstered against ostensible claims of technological efficiency.

Our paper is organized as follows: Initially, we describe the advancements of algorithm-based HR decision-making that provide measures for organizations to monitor their employees. Next, we suggest that algorithm-based HR decision-making is neither as objective nor as morally neutral as it is often portrayed. Then, we argue that algorithm-based HR decision-making as marketed by technology companies supports the implementation of quantitative indicators and compliance mechanisms at the expense of employees’ personal integrity. Finally, we suggest four mechanisms, namely critical data literacy, ethical awareness, the use of participatory design approaches (i.e., defined as a methodology to include future users in the implementation process, Van der Velden and Mörtberg 2015), and private regulatory regimes within civil society to reduce negative consequences of algorithm-based decision-making.

## A Brief History of Algorithm-Based HR Decision-Making

Attempts to gather information about workers and to create transparency regarding workplace behavior are by no means new phenomena (Ananny and Crawford 2018; Garson 1989; Rule 1996). Indeed, they can be traced back to philosophers, such as Adam Smith and Jeremy Bentham (Rosenblat et al. 2014). Bentham’s idea of the *Panopticon* has been influential not only on philosophers, such as Foucault (1977), but also on management theorists (Ball 2010; Fox 1989; Zuboff 1988). It is routinely being invoked by surveillance critics and critical algorithm scholars to this day (Galič et al. 2017; Introna 2015). At the turn of the twentieth century, management theorists, such as Frederick Taylor, based their productivity experiments on the assumption that unobserved workers are inefficient, which introduced the need for constant performance monitoring (Saval 2014). Following Ball and Margulis (2011), we understand the terms “workplace monitoring” and “workplace surveillance” synonymously, as both terms “denote similar practices, namely the collection and use of data on employee activities in order to facilitate their management.” However, in our manuscript we use the term workplace monitoring as it has a less value-laden and more neutral and connotation than surveillance.

A first step toward algorithm-based HR decision-making was the introduction of electronic performance monitoring during the last decades of the twentieth century. Electronic performance monitoring includes, for example, automated tracking of work times as well as internet-, video-, audio- and GPS-based observation of employees on the job (Stanton 2000). Alder and Ambrose (2005) estimated that this type of control affects between 20 and 40 million U.S. workers. Electronic performance monitoring is traditionally geared toward standardized jobs, targeted explicitly and mostly overtly to monitor job-related behavior, task performance, and compliance with company rules (Ball 2010). Yet, current algorithm-based HR decision-making tools go far beyond the monitoring activities described in the electronic monitoring literature (Ananny 2016; Dourish 2016; Seaver 2017).

Recent applications of algorithm-based HR decision-making differ from traditional electronic monitoring in at least three ways (Beer 2017; Weibel et al. 2016; Zarsky 2015). First, in addition to performance data, current algorithm-based decision-making tools also monitor contextual (not task-related) performance, such as employee engagement and overall health, as well as employee behavior outside of the workplace. Furthermore, current algorithm-based HR decision-making tools are increasingly able to exploit novel types of data, such as internet browser histories, keystrokes, electronic calendars, and location data from wearable devices, such as fitness wristbands and mobile phones (Angrave et al. 2016; Rosenblat et al. 2014). Thus, organizations can monitor employees’ private activities in many ways, including on their Facebook accounts (Angrave et al. 2016); they can also collect health-related information, such as employees’ fitness data, to superimpose health screening programs (Rosenblat et al. 2014). Furthermore, firms may trace their employees’ moods by using video-based facial recognition techniques or by analyzing the content of email messages (Angrave et al. 2016). HR managers could use these novel data sources, for example, to create more fine-grained measures to evaluate employees’ motivation, training needs, and healthiness. Currently, the statistical tools to analyze data from large, unstructured sources are emerging in contemporary HR information systems.

Second, current algorithm-based HR decision-making tools can integrate data from a variety of sources traditionally kept separate. Key players in the field of HR information systems, such as Oracle, IBM, and SAP, for instance, offer integrated talent management software packages to collect data from a range of existing databases (Angrave et al. 2016). More and more, different sources are being grouped together to create consolidated profiles of employee data. Typically, data held in such HR information systems are composed of information on the employees hired, a person’s pay, and hours worked and, depending on the job, various performance-related measures. On top of integrating HR reporting systems and electronic devices, current HR information systems are increasingly linked with other organizational resource planning software units involving aspects such as customer relationship management and manufacturing management, supply chains, logistics, accounting, and finance (Angrave et al. 2016). Integrating those different data sources offers the promise for HR managers to generate measures for employee performance based on less obtrusive data than before.

Finally, the technical capability of algorithms to meaningfully analyze data has largely expanded (Amoore and Piotukh 2015; Ananny and Crawford 2018; Dourish 2016). According to common classification in management (Davenport 2013; Souza 2014), algorithms can be broadly divided into three categories: descriptive, predictive, and prescriptive algorithms.

First, *descriptive algorithms* aim at analyzing what happened in the past and how this influences the present. Descriptive algorithms show, for example, the distribution of variables or the association between variables. Descriptive algorithms are build on relatively simple statistics, such as means, standard deviations, correlations, or percent changes. A typical example of using descriptive algorithms in HR is a balanced scorecard. This is a common performance management tool to keep track of strategically relevant indices, such as absences, turnover, and supervisor performance feedback (Davenport 2013; Souza 2014). In the context of algorithm-based HR decision-making, descriptive algorithms can become very powerful due to the increasing granularity of such data and their integration from different sources. Such algorithm-based HR decision-making applications allow users to plot employees’ informal social networks (e.g., using email, Bluetooth, video, or GPS data) or examine the relationship between service employees’ mood and customer satisfaction rates (e.g., by correlating results of video-based facial recognition with customer satisfaction ratings). An HR example based on such a descriptive algorithm is Microsoft’s software Yammer. Yammer is a blogging platform that helps employees coordinate activities and share documents across organizational subunits. One of its features is an emotion recognition software called Crane that analyzes feelings workers express in messages posted to a Yammer company network. Crane also displays employees’ emotions over time, using a line graph to show the aggregated levels of excitement, confusion, and other feelings at the subunit or firm level (Simonite 2012). Additionally, Crane provides managers with the topics or words most often associated with those feelings, offering managers a relatively easy-to-handle HR instrument with the potential to track employees’ mood.

Descriptive algorithms can help HR managers track employees’ motivation, measure their performance, generate profiles of desired job candidates, and identify important strategic topics that can create either anxiety or excitement among employees.

Second, *predictive algorithms* are used to forecast what might be the result of certain past- or real-time observations on future outcomes. Predictive algorithms determine the likelihood of such outcomes (or situations) to occur. Applied methods are advanced regression techniques, machine-learning algorithms, and data mining approaches (Davenport 2013; Souza 2014). Typically, predictive algorithms provide a score that represents the probability of an event to occur. An example of a predictive algorithm is fraud prediction. JP Morgan, for instance, uses an application to identify potential future rogue traders by relying on an algorithm that analyzes multiple data points, such as whether employees skip compliance classes, violate personal trading rules, or breach market-risk limits (Son 2015). Another example is a recruitment algorithm developed by the technology firm Xerox Services. This algorithm works as an advanced support system for hiring staff in Xerox’s call centers by offering a score of how well the applicant would fit the job (Peck 2013). The algorithm behind this HR tool analyzes data provided by applicants via an online application tool and offers a cognitive skill assessment, personality test, and multiple-choice questions to see how well the applicant would deal with specific challenges on the job. Teri Morse, vice president of recruiting at Xerox Services, stated that the company was “getting to the point where some of our hiring managers don’t even want to interview anymore” because they would rather rely on the scores provided by the software (Peck 2013).

Predictive algorithms can provide compliance officers with suggestions of suspicious employee behavior for which these officers then investigate in detail whether this behavior is in line with the firm’s compliance regulations. Furthermore, predictive analytics help HR managers recruit employees. For an HR manager, however, the perceived objectivity and unbiased nature of the algorithm makes it difficult to recruit a different person than the one suggested by the predictive algorithm.

Finally, *prescriptive algorithms* aim at delineating what should be done in light of different possible scenarios. Prescriptive algorithms go beyond forecasting future outcomes by also suggesting different courses of action to benefit from alternative scenarios and demonstrating the consequences of each possible decision (Davenport 2013; Krumeich et al. 2016; Souza 2014). Prescriptive algorithms stem from the academic subfield of operations research and are based on similar methods as predictive algorithms; however, they add simulations and scenario-based techniques to the repertoire (Stewart and McMillan 1987). One HR-related example stems from the logistic firm UPS. UPS uses an artificial intelligence technology to shorten parcel delivering routes, thereby saving time and fuel (Konrad 2013). To do so, UPS equipped its parcel delivery cars with sensors, registering all brakes and turns as well as the car users’ personal driving habits. These data are matched in real time with other data, such as weather and traffic news. UPS does not only use the results to make the driving routes more efficient, but it also uses these results as part of the key performance indicators to rate their drivers’ performance (Zax 2013).

Prescriptive algorithms can be used to improve the efficiency of employees’ behavior, such as in the UPS example, or to model complex strategic HR decisions. Given the complexity of these decisions and the vast number of variables molded into such an analysis it becomes virtually impossible for a human being to understand exactly how the algorithm proceeded and how it modeled information into a decision (Ananny 2016; Neyland 2015; Stohl et al. 2016). Prescriptive algorithms can broadly serve two functions—decision support and decision automation. The strategy example related to decision support and the UPS example refers to decision automation where, during the normal operational procedure, no human being is involved in the decision-making process.

The three types of algorithms—descriptive, predictive, and prescriptive—offer increasing analytical power. But with increasing analytical power, these algorithms also become more opaque regarding their underlying hidden assumptions (Burrell 2016; Pasquale 2015; Zarsky 2015).

## Algorithm-Based Decision-Making: Objective, Unbiased, and Efficient?

In the last section, we explained how the interplay between algorithms and data may enable a wide range of monitoring techniques to create more transparent and efficient HR processes. In the following section, we propose two important characteristics influencing how algorithm-based HR decision-making is implemented into organizations: First, an algorithm-based decision is neither as objective nor unbiased as portrayed by its proponents (Bilić 2016; Porter 1996; Ziewitz 2015). Second, algorithm-based HR decision-making is embedded in a particular “worldview” (Lowrie 2017; Zuboff 2015) related to its makers and funders. We suggest that these characteristics make it difficult for HR managers to implement algorithm-based HR decision-making in ways that do not hurt employees’ personal integrity.

### The Assumption of Algorithm’s Objectivity

Discussions of algorithm-based decision-making often invoke a “mythology” centered on objectivity (Amoore and Piotukh 2015; boyd and Crawford 2012; Ziewitz 2015). Technology firms suggest, for example, that algorithm-based HR decision-making increases efficiency, enables fact-based decision-making, reduces particularism, and offers solutions to talent shortage (Porter 1996). An area where algorithm-based HR decision-making techniques could become particularly important is recruitment. In industries with a high fluctuation, such as retail or hotel chains, firms must scan a vast number of resumes per year and conduct a large number of interviews. In such a context, algorithm-based HR decision-making techniques could be helpful in reducing manual, labor-intensive processes. Providers of recruitment algorithms, for example, promise that “when using an automated process, all candidates are screened against the same criteria consistently” (Why 2018). Vendors of HR tools promise that the results of algorithms are fairer and less biased than human judgment. Accordingly, those firms advertise the resulting staffing solutions as a means to help firms win the war for talent (Delle Donne 2017). Most prominently, technology firms propose that algorithm-based HR decision-making is evidence-based, bias-free, and superior to human intuition.

Recent literature has questioned the promises of algorithms’ objectivity (Bilić 2016; Porter 1996; Thelwall 2018). For example, O’Neil (2016) has suggested, drawing from her own experience as a mathematician in finance, that an algorithm used for HR processes, such as employee recruitment, evaluation, or performance appraisals, are still impaired by racial and gender biases. For machine-learning algorithms, these biases can also stem from the data with which the algorithm was trained. An algorithm trained on historic employment data, for example, would integrate that most managers are male, thereby assuming that women are less interested in management positions. Consequently, this recruitment algorithm would not show an employment advertisement for a management position via social media to women. The advertisement would, in fact, be invisible to women; thus, women would have no opportunity to apply. In this case, a recruitment algorithm might be actively reifying the original gender bias, based on the data with which it was trained (Devlin 2017; O’Neil 2016). Similarly, Buolamwini and Gebru (2018) showed in a recent study that facial recognition algorithms still exhibit significant racial bias, as these algorithms are less able to detect the gender of African-American women than of Caucasian women. Accordingly, Noble (2018) found that internet search algorithms privilege whiteness and discriminate against African-Americans, particularly against African-American women. The key issue here is that developers of machine-learning algorithms use data to train their algorithms. As these data might be biased according to an external reference point, the training has the great capacity to be faulty (Barocas and Selbst 2016; Martin 2018).

An additional reason why racial and gender stereotypes may persist is that the predominant code of algorithms tends to reflect the cultural background of its developer(s) (Lowrie 2017; Seaver 2017; Striphas 2015). In other words, algorithms implicitly target audiences similar to their creators. Therefore, Crawford (2016, p. 1) infers that since the majority of artificial intelligence is created by Caucasian males, the algorithms would thus reflect “white guy” influences. Making this impact more notable is the understanding that subtle forms of discrimination are often difficult to detect. Algorithms in the context of HR decision-making are typically black boxes based on proprietary code that technology companies are not willing to share with the public (Burrell 2016; Pasquale 2015). This lack of transparency makes it difficult for HR managers to uncover biases either in the code of an algorithm itself or in the data with which the algorithm was trained (Martin 2018).

Furthermore, this lack of transparency has inspired calls in academia and practice to hold algorithms accountable (Ananny and Crawford 2018; Angwin 2016; Diakopoulos 2016; Neyland 2015). One example is Stanford University’s artificial intelligence laboratory’s AI4ALL (Artificial Intelligence for ALL), which addresses transparency problems in algorithms (AI4ALL 2018). Similarly, technology firms have become more aware of this topic after several scandals. This was illustrated by Google (2017), which published videos to raise ethical awareness for the lack of transparency in algorithms. This awareness is vital, as biases can impede the objectivity of any HR-related practice, such as employee recruitment, evaluation, or performance appraisals.

### The Underlying Values of Algorithms

Zuboff (2015) argued that there is a specific Silicon Valley culture that puts forward algorithm-based decision-making. Algorithms reflect the norms and values of its makers and funders (Crawford 2013a; Hallinan and Striphas 2014; Jasanoff 2016). Hence, Zuboff (2015) has argued that knowing the Silicon Valley belief system is necessary to understand underlying logic of algorithm-based decision-making. Barbrook and Cameron (1996, p. 44) described Silicon Valley’s entrepreneurial culture as a “Californian Ideology,” that “promiscuously combines the free-wheeling spirit of the hippies and the entrepreneurial zeal of the yuppies. This amalgamation of opposites has been achieved through a profound faith in the emancipatory potential of the new information technologies. In the digital utopia, everybody will be both hip and rich.”

Traditionally, white affluent men have developed and funded the technological advancement of algorithms (Crawford 2016; Thomas et al. 2018; Watson 2016). Hence, it is not surprising that the algorithms these men created might also represent their worldview (Seaver 2017; Striphas 2015; Zuboff 2015). Morozov (2013, p. 1) proposed that the culture of Silicon Valley is poised by a belief system of “solutionism.” This solutionism is mirrored by technology firms’ marketing claims, promising notions of technological mastery, control and innovation, and portraying technology as a somewhat independent actor that produces reliable, sustained technological progress (Dovey and Kennedy 2006; Turkle 1995; Zuboff 2015). According to Morozov (2013, p. 1), the culture of Silicon Valley reflects an “intellectual pathology that recognizes problems as problems based on just one criterion: whether they are ‘solvable’ with a nice and clean technological solution … and not because we’ve weighed all the philosophical pros and cons.” In sum, we suggest that algorithm-based HR decision-making tools might be biased and reflect the belief system of their developers and entrepreneurs. In the next chapter, we will discuss how these threats may harm employees’ personal integrity.

## Ethical Challenges to Employees Personal Integrity

Following Möhlmann and Zalmanson (2017), we suggest that the novel challenges posed by algorithm-based HR decision-making go beyond factors that have already been described by the personal integrity literature, factors such as rigid organizational structures or employees’ own self-interested behavior (Adler and Borys 1996). Even before the advent of big data, institutional structures of HR practices have partly compromised the delicate balance between integrity and compliance (Wilcox 2012). However, recent research in the context of the sharing economy suggests that algorithm-based decision-making may amplify these tensions. For example, in a case study of Uber drivers, Rosenblat and Stark (2016) found that algorithm-based decision-making increases information asymmetries between management and drivers and decreases the drivers’ experience of control. This results in more negative feelings by the drivers toward the company. Accordingly, in another case study with Uber drivers, Möhlmann and Zalmanson (2017) showed that algorithm-based decision-making reduces a driver’s sense of autonomy. As a response, drivers start to resist and manipulate the decisions made by the algorithm. Furthermore, Lee (2018) found in an experiment that when recruitment decisions and performance evaluations are made by an algorithm they are less likely to be perceived as fair and trustworthy, while simultaneously evoking more negative emotions than human decisions. As such, algorithm-based HR decision-making may not only aggravate already known quandaries for personal integrity (Ekbia et al. 2015) but also may generate novel tensions on the balance between compliance and integrity.

In her seminal paper, Paine (1994) defines *integrity* as a “concept of self-governance in accordance with a set of guiding principles.” Integrity is often contrasted with compliance. Compliance is organizationally governed behavior, i.e., making employees conform to (organizational) standards and rules by means of monitoring as well as by sanctioning and incentivizing rule conformity. While the integrity approach is broader, deeper, and more demanding than a mere focus on compliance, compliance and integrity are generally assumed to complement one another. Yet, despite the fact that integrity has been discussed in management literature for at least five decades, a shared understanding has not yet been established (Palanski and Yammarino 2009; Parry and Proctor-Thomson 2002). Hence, some scholars regard the concept of integrity as vague and ill-defined (Rieke and Guastello 1995). Drawing from the inconsistent literature, we thus distinguish between two concepts of integrity: moral integrity and personal integrity. We intend to address both concepts briefly but will focus on personal integrity in this paper.

Moral integrity can be loosely defined as coherence between moral convictions and behavior. Moral integrity takes into account that shared moral values play an important role for integrity (McFall 1987). From this point of view, being a person of integrity means promoting and committing to certain moral values (e.g., equality, self-determination) and condemning certain actions or practices considered to be negative (e.g., corruption, fraud, opportunism). In business, this also implies committing to an organization’s guiding values. The “composites of personality traits” (Becker 1998) connected to a moral notion of integrity reflect a myriad of possible dimensions of an actor’s morality (Tomlinson et al. 2014). Hence, according to discourse ethics these aspects of morality are difficult to prescribe because most values are context depended and “the validity of moral claims cannot be justified by an isolated individual reflecting monologically upon the world but can be validated only intersubjectively in argumentation processes” (Scherer 2015, p. 499). As every organization needs to determine its own guiding principles, it is difficult to establish a universal definition of material ethics values. Therefore, in this paper, we will not attach specific moral values to the general notion of moral integrity as a concept.

The concept of *personal integrity* is pivotal to our examination; it can be defined as an individual’s consistency between convictions, words, and actions (Palanski and Yammarino 2009). This is also implied by the Latin origin of the term: ‘integritas,’ i.e., being whole or undivided. In accordance with Bauman (2013), we view personal integrity as a non-moral notion of wholeness, similar to Simons’ (2002) understanding of integrity as “the perceived pattern of alignment between an actor’s words and deeds.” In other words, personal integrity is about “walking the talk.” However, as the concept of personal integrity merely takes into account whether a person acts in accordance with their convictions, an ethical evaluation of such convictions is still necessary. For instance, a manager who ideologically equates shareholder value with corporate responsibility may be considered a person of integrity even if his convictions may be unreasonable or problematic from an ethical point of view. In this instance, his moral integrity would be judged rather negatively.

Hence, personal integrity is needed as a baked-in compass as employees are required to hold themselves accountable to the standards they have set for themselves based on their individual convictions and values. Such self-regulation, however, implies autonomy and self-determination as a prerequisite (Weibel 2007). Yet, self-determination is jeopardized by algorithm-based HR decision-making tools through three avenues: (1) diminishing opportunities for human sense-making, (2) tendency to rely on technology in situations where reflexivity would be needed, and (3) a lack of moral imagination.

### Algorithm-Based Decision-Making Marginalizes Human Sense-Making

Proponents of algorithm-based decision-making hope to change the corporate environment “from a culture that largely depends on heuristics in decision-making to a culture that is much more objective and data driven and embraces the power of data and technology,” as proposed in a recent McKinsey study (Buluswar et al. 2016, p. 1). The lures and the pressure to implement corresponding tools are tremendous and result from a demand for more efficiency, an increase in rationality, and fewer human errors. Integrating algorithm-based HR decision-making tools into formal and informal decision-making processes within organizations is not a mere technical issue. Instead, it is the result of a complex relationship between data, analytical tools, and human sense-making (Sharma et al. 2014), defined as an “ongoing retrospective development of plausible images that rationalize what people are doing” (Weick et al. 2005, p. 409). As such, human sense-making is an experience-based search for the “story” behind organizational circumstances. Human sense-making is important for organizational functioning because it helps those impacted deal with everyday ambiguity, settling for plausibility, and rationalizing one self’s and others’ behavior and actions (Weick et al. 2005). As such, personal integrity plays a pivotal role because an individual’s personal convictions feed into a collective organizational sense-making process that Taylor and Van Every (2000, p. 275) described as a “way station on the road to a consensually constructed, coordinated system of action.” As such, algorithm-based HR decision-making can be either a catalyst or a challenge to the quality of the human sense-making process.

On the one hand, algorithm-based decision-making can enhance human sense-making because it can help make decisions more rational, more fact-driven, and more reliable. Descriptive algorithms, in particular, provide an increase in the amount of information, usually without stipulating interpretation patterns.

On the other hand, delegating the interpretation and evaluation of data to analytics software challenges human sense-making, as algorithms increasingly prescribe supposedly desirable outcomes and sometimes implicitly or even explicitly recommend courses of action (Mittelstadt et al. 2016). This certainly applies to prescriptive algorithms, which deduce decisions from their interpretation of the data. The ever-increasing ‘datafication’ of business brings with it the hope of “turning that data into something of value” (Lycett 2013), which is a challenging and complex process. Because data do not speak for themselves, it is up to humans to make sense of analytics results, to contextualize and interpret them, and to consider the consequences of algorithmic decisions beyond the scope of what machines have been trained to do for specific purposes. The more prescriptive algorithmic decisions become and the broader the scope of their decisions, the higher the risk of humans being crowded out. This would also include their moral convictions. Human convictions and decisions are increasingly confronted with the “widespread belief that large data sets offer a higher form of intelligence and knowledge that can generate insights that were previously impossible, with the aura of truth, objectivity, and accuracy” (boyd and Crawford 2012).

This trend toward prescriptive analytics puts pressure on individuals not to rely on their specifically human skills, such as critical reasoning, emotions, and intuitions, but instead to put all their trust in the supposedly neutral and superior decisions made by algorithms. It also challenges organizational sense-making processes and routines that, up until now, allowed individuals to maintain personal integrity by interacting with one another at eye level and discussing their convictions and deeds in a non-hierarchical manner. The appreciation for such human encounters comes under siege when algorithms are being marketed as infallible compared to volatile, emotional, and deficient human beings.

### Algorithm-Based Decision-Making Underscores Blind Trust in Rules

Whether we like it or not, human action often leads to human error. Human errors frequently result from issues such as oversight, intrinsic human decision biases, conflicting interpretations of information, and opportunistic behavior. All of these issues are seen as shortcomings not prone with algorithm-based HR decision-making. Algorithm-based decisions are often expected to be objective because they remove irrelevant sociocultural constraints from the equation (Parry et al. 2016). Therefore, in line with the worldview described in the last section, the U.S. technology community views human reasoning capacities as inferior to those of ever-improving machines. In fact, this has been a matter of a public discussion in recent years, as several private initiatives and publications have addressed concerns over the singularity, i.e., the point at which machines become too smart for humankind to maintain control over its own fate (Boström 2014). But even if we remove the underlying notion of dystopian science fiction from this line of reasoning, it is difficult to deny that the assumption of machines being superior to human reasoning and moral convictions, leading to an overly strong belief in rules and the ability to produce predictable outcomes.

Scholars have investigated and discussed this effect of technology-based decision-making for years. Kottemann et al. (1994), for example, have examined computer-based decision aids with a relatively simple structure. The authors have shown that these programs engender an “illusion of control” which causes decision-makers to overrate their effectiveness, resulting in inflated performance estimations (Kottemann et al. 1994, p. 33). In turn, Van Dijck (2014, p. 198) has suggested that the recent technological changes around big data are accompanied by an “ideology of dataism,” emphasizing a belief in the “objective quantification and potential tracking of all kinds of human behavior.” According to Van Dijck (2014, p. 198), this development is associated with a continuous monitoring using data which the author refers to as “dataveillance”.

Taylor (2007) expresses the experience of being overwhelmed by seemingly superior rules implying (blind) faith due to their very superiority as follows:

“… the ‘code fetishism,’ or nomolatry, of modern liberal society is potentially very damaging. It tends to forget the background which makes sense of any code: the variety of goods which the rules and norms are meant to realize, and it tends to make us insensitive, even blind, to the vertical dimension. It also encourages a ‘one size fits all’ approach: a rule is a rule.”

Even though Taylor addresses religion, and not big data or algorithm-based decision-making, the concept of nomolatry does give us an ideal parallel with which to work. Assuming that algorithms are more accurate than humans, users of analytics tools simply cannot explain or even retrace the reasons for algorithmic recommendations. Since algorithms are presumably superior, it is difficult to argue against such recommendations (Thomas et al. 2018). Thus, the more complex a decision, the more tempting it is to believe in the superiority of algorithms. After all, what better way to explain a potentially risky decision to colleagues, management, or shareholders than by pointing to the highly sophisticated (and expensive) algorithm-based HR decision-making tool purchased by the company to be used for such a situation? In order not to be held accountable for human error, humans might willingly subject themselves to the nomolatry and automation bias imposed by algorithmic decision-making. As Taylor (2007) points out, one possible victim of this process is the “vertical dimension,” which is to say, our willingness to trust others and to engage in discourse with them. Nomolatry and a naive belief in the superiority of data-based decisions, thus, can go hand in hand, undermining trust and discourse between people within organizations, and replacing integrity as well as trust in human capacities with a strong emphasis on trust in technology-based systems, compliance, and risk avoidance.

At the same time, the belief in the infallibility of the machine or at least the greater resistance to machine errors is extremely risky, as O’Heigeartaigh (2013, p. 1) suggested:

“Human decision-making is riddled with biases and inconsistencies, and can be impacted heavily by as little as fatigue, or when we last ate. For all that, our inconsistencies are relatively predictable, and have bounds. Every bias we know about can be taken into account, and corrected for to some extent. And there are limits to how insane an intelligent, balanced person’s ‘wrong’ decision will be … This is not necessarily the case with machines. When a machine is ‘wrong,’ it can be wrong in a far more dramatic way, with more unpredictable outcomes, than a human could.”

Human errors often trigger learning processes and, thereby, may enable individuals to find the right, value-consistent answer to complex problems. This learning process is an important part of personal identity and self-regulation as it both enlarges the action repertoires of individuals, giving them more options for expressing their self-determination, but also enables personal growth, which is also linked to integrity (Ryan and Deci 2000). Hence, errors can trigger organizational learning processes that may actually strengthen integrity in the long run. Also, as already elaborated on, machines are by no means bias-free. They may threaten integrity at the organizational level, as legal and moral accountability are difficult to determine in the complex interplay of humans and machines.

There is currently no reason to assume that blind faith in algorithm-based decision-making has reached a level comparable to Taylor’s idea of nomolatry. As the aforementioned KPMG survey (2015, p. 8) highlights, a majority of executives, most of them working in an HR function, remain skeptical of a possible benefit for the HR function. At the same time, however, there is a strong social pressure to apply those techniques, due to the fact that big data has a prominent place in the popular press and organizational leaders might feel like everybody else in the business world is using such techniques. Hence, the overwhelming majority of those skeptics is arranging for a short-term expansion of big data and advanced analytics, even though skills, resources, and experience regarding analytics are still lacking. Eventually, this may lead to a situation where faith in the system is the only way to reduce (or avoid) the technological complexity; “algorithm fetishism,” as Taylor puts it, may become a convenient and tempting option.

### Algorithm-Based Decision-Making Lacks Moral Imagination

Dealing with moral dilemmas, finding new approaches to solve novel problems and the well-tried “thinking outside the box” presupposes moral imagination. Especially in business organizations, moral uncertainty and moral complexities are increasing, which leads to a higher value divergence, increasing moral disagreement, resulting in more moral conflict than before (Gutmann and Thompson 1996). This process of dealing with moral conflicts requires the ability to compromise. Goodstein (2000) suggests that such moral deliberation is tied to the ability of enacting moral imagination. As such, an organizational context that facilitates moral imagination also serves as a prerequisite for sustaining and developing personal integrity. Werhane (1999) defines moral imagination as:

“the ability in particular circumstances to discover and evaluate possibilities not merely determined by that circumstance, or limited by its operative mental models, or merely framed by a set of rules or rule-governed concerns. In management decision-making, moral imagination entails perceiving norms, roles, and relationships entwined in any situation. Developing moral imagination involves heightened awareness of contextual moral dilemmas and their mental models, the ability to envision and evaluate new mental models that create new possibilities, and the capability to reframe the dilemma and create new solutions in ways that are novel, economically viable, and morally justifiable.”

However, algorithm-based leadership and motivational tools are limited in regard to qualifying as well as intrinsically motivating employees for moral imagination. Algorithm-based HR decision-making, no matter how sophisticated, cannot compete with the compelling storytelling techniques (Forster et al. 1999; Parry and Hansen 2007) and emotional sincerity (Gardner et al. 2009) of successful human leaders. Employees’ motivation and commitment are inspired by leaders’ personal integrity (Calhoun 1995) and self-determination (Weibel 2007), whereas algorithm-based decisions both lack this human trait and prevent self-determination. Accordingly, Johnson (1994) has characterized any moral understanding as fundamentally imaginative—this is hardly compatible with algorithms that might have the potential to learn and improve, but are still bound to limiting parameters, such as their training data and strict objective.

This lack of moral imagination is problematic because algorithms make decisions within defined parameters and under restrictions, following reductionist principles (Bhattacharya et al. 2010). They are thus unable to operationalize qualitative criteria and to think outside the box. Ethically challenging scenarios that require creativity, e.g., to solve dilemmas, are problems beyond the realm of what analytics tools can solve. This becomes problematic whenever prescriptive analytics software suggests a course of action, implying there is no alternative. In such a case, personal integrity is especially important for interventions that confront the alleged superiority of the machine to perceive and correct an error.

## Balancing Tensions Between Compliance and Integrity

Algorithm-based HR decision-making seems to be an “unstoppable force” (KPMG 2015, p. 28) and its trail of success might be a foregone conclusion. As such, algorithm-based HR decision-making increasingly provides authoritative answers according to which goals, standards, incentives, and sanctions are created. Increasingly, algorithm-based HR decision-making influences, predetermines, and may even replace human decisions within organizations, potentially compromising the integrity of all parties involved. Thus, the logic of algorithm-based decision-making lends itself very well to the “compliance approach to ethics” (Paine 1994), which makes rather pessimistic assumptions about human behavior and emphasizes control and sanctions. This comes at the expense of employees’ agency and integrity, as algorithm-based decision-making can be used as an instrument of centralized formal managerial control, emphasizing extrinsic motivation instead of supporting intrinsic motivation based on values and moral convictions (Weibel 2007; Weibel and Six 2013).

Ideally, however, compliance and integrity should complement each other. Compliance provides institutional support and a set of agreed-upon norms that employees can refer to in complex and challenging situations that require boundaries, while organizational integrity in the form of moral values and moral behavior signals the ethical stance of the organization. In doing so, organizations protect their members from being left alone when faced with complex moral challenges. Yet at the same time, individuals also need a certain degree of autonomy to practice and live their personal integrity—to be able to creatively work on solutions that are both economically and ethically sound. Therefore, a balanced approach must also allow for self-determination among other things, including the ability and opportunity to foster organizational discourses and participation. A healthy balance between compliance and integrity is thus “option-excluding” and “discourse-opening” at the same time (Rasche and Esser 2007).

Algorithm-based decision-making jeopardizes this delicate balance. An organization’s discourse culture will be at risk if black box algorithms take over the decision-making process, potentially demoting the humans from the position of decision-makers to mere decision-announcers. But Parry, Cohen, and Bhattacharya (2016) argue that this could be advantageous when it comes to unpalatable decisions: as the case for those might be made more convincingly and more transparently by a neutral, de-individualized source, as this might be perceived as more convincing or transparent. This suggests that an algorithm-based decision may feel more legitimate than a human one simply because it is difficult to question the reasoning process of a black box. However, this would stand in stark contrast to the integrity approach, resulting in a “culture of silence” (Verhezen 2010) in which employees are disincentivized from calling out questionable practices based on the employees’ moral convictions, as an algorithm is not an accountable entity with which one could reason (Angwin 2016). Based on this analysis, we are concerned that relying too heavily on algorithm-based decision-making may lead to a compliance-oriented ‘command and control’ culture within organizations, where discourse is replaced by de-individualized, de-humanized, and de-socialized decisions of algorithms.

Despite this rather grim outlook, formal control may yet be useful in supporting an integrity-based culture of trust within organizations, but only when certain conditions are met. In this context, Weibel (2007) as well as Weibel and Six (2013), emphasize the vital role of individual autonomy, which is expressed mainly in participatory decision-making processes. Furthermore, they stress the importance of honest, learning-oriented, and constructive feedback mechanisms, as well as a holistic appreciation of work performance. These factors will become increasingly important in order to maintain a balance between compliance and integrity, as algorithm-based decision-making tends to overemphasize quantifiable targets and quantitative indicators (Parry et al. 2016). Thus, while algorithm-based decision-making promises to make good on Taylorist ambitions, removing the unpleasant messiness of human experience and conflict within organizations, it may lead to a data-driven, performance-oriented, and overly compliance-focused organizational culture in which there is little room for moral autonomy and integrity. This turns employees into mere bystanders of algorithmic decision-making. In the final section, we will make suggestions on how to lessen these detrimental effects on personal integrity.

## Cushioning the Detrimental Effects of Algorithm-Based HR Decision-Making

Up to this point, we have shown how algorithm-based HR decision-making provides methods for organizations to monitor their employees. Furthermore, we have demonstrated that algorithm-based HR decision-making exhibits potential biases and is embedded into an organization’s culture, which can manifest itself in an illusion of control (Durand 2003). Then, we explained how the quantitative logic of algorithm-based HR decision-making may impair employees’ personal integrity. We suggest that potential negative effects of algorithm-based HR decision-making can be addressed at the individual level of analysis (e.g., at the level of HR managers and employees) as well at the organizational level of corporate actors. Furthermore, possible solutions may consider the interplay between those levels. In this final section, we offer an outlook of how critical data literacy, ethical awareness, the use participatory design methods, and private regulatory regimes of actors from the civil society may dampen the negative effects of algorithm-based HR decision-making.

First, critical data literacy can support managers and employees in navigating the cultural and ethical complexities around algorithm-based HR decision-making (Bhimani and Willcocks 2014). Organizational members should understand the social embeddedness of technology and develop critical thinking skills—on top of their technical expertise. These critical skills will take time to make their way into organizations, as the phenomenon of algorithm-based HR decision-making is still relatively new. Academic institutions have not yet had much of a chance to teach the critical reflection skills necessary to identify biases in algorithms and underlying taken-for-granted assumptions. However, scholars in the fields of critical algorithm studies, digital ethics, and business ethics have already generated knowledge that could help create critical data literacy among managers and (other) employees (Martin and Freeman 2004; Nijhuis 2017). Furthermore, diversity initiatives can help diminish biases related to algorithms because a broader representation of viewpoints can reduce the number of blind spots (boyd and Crawford 2012; Crawford 2013). The technology firm Intel, for example, had spent three hundred million dollars to improve its gender and racial diversity (Vara 2015). This way, vendors of algorithm-based HR decision-making might also increase their own critical data literacy. Despite all organizational efforts and shortcomings, building critical data literacy remains essentially an intersectional challenge that must be tackled within different institutional settings such as schools, universities, and business organizations.

Second, ethical awareness might help organizational members in dampening algorithm-based HR decision-making’s detrimental consequences. Such awareness is particularly important for HR managers in implementing such algorithms. As part of ethical awareness, organizational members can learn to critically assess algorithm-based HR decision-making’s opportunities and limitations. Furthermore, organizational members should jointly engage in discourse of how algorithm-based decision-making can be applied within an organization. Technology firms have an interest in not making their algorithms’ code and training data transparent. This is a challenge for HR managers, as it hinders them from completely understanding the algorithm-based HR decision-making tools they may want to purchase. Nevertheless, HR managers should critically reflect what kind of algorithm-based decision-making tools they need. Not every algorithm needs to maximize monitoring and data extraction, and not everything that can be accomplished technologically is ethically legitimate. To increase ethical awareness, organizations may also install a board of ethics that help HR managers decide on the appropriateness of implementing certain algorithm-based decision-making solutions. Increased ethical awareness may offer several benefits for organizations. At present, governments are increasingly tightening their data-protection laws (Ajunwa et al. 2017). Ethical awareness might help prohibit costly lawsuits and prevent a loss of reputation. In fact, an organization willingly limiting its data extraction efforts might generate a competitive advantage by attracting top talent and improving its image (Hasselbalch and Tranberg 2016; Martin 2016).

Furthermore, moral imagination is particularly conducive to initiating a self-critical, reflexive process in organizations, because moral imagination helps anticipate the perspectives and moral concerns of third parties (Werhane 1998). Algorithms can cope with quantifiable phenomena, yet still struggle to deal with qualitative questions and normative controversies (Bhattacharya et al. 2010). Moral imagination lends itself well to challenge this quantitative logic of algorithms, modify the scripts of human behavior implied by algorithms (Verbeek 2006), and offer organizational members guidance on how to behave appropriately in specific situations (Vidaver-Cohen 1997). To this aim, corporate actors can create spaces for discourse and reflection (Rasche and Esser 2007) that are not subordinate to the quantitative logic of algorithms. This endeavor will not be trivial, as the idea of algorithm-based leadership decision-making without a human-held veto already looms around the corner of the current debate (Parry et al. 2016). Nevertheless, organizations can cultivate and encourage both ethical awareness and moral imagination through role modeling of the leaders and communicative and regulative structures (such as codes of conduct, trainings, and policies).

Third, participatory design methodologies may help HR managers implement algorithm-based HR decision-making tools in a way that does not harm employees’ personal integrity. Participatory design is “a design methodology in which the future users of a design participate as co-designers in the design process” (Van der Velden and Mörtberg 2015, p. 11). Its guiding principles include equalizing power relations, implementing democratic practices and mutual learning, among other things. Value-sensitive design is a type of participatory design that lends itself particularly well to addressing the ethical challenges of algorithm-based HR decision-making. It is “a theoretically grounded approach to the design of technology that accounts for human values in a principled and comprehensive manner throughout the design process” that is based on an “integrative and iterative tripartite methodology, consisting of conceptual, empirical, and technical investigations” (Friedman et al. 2013, p. 348).

As part of the design process, HR mangers should take employees seriously as human beings and not just as data objects. This requires HR managers to recognize that employees are not mere bystanders or even data bodies, but instead partners in a mutual process. This understanding would open avenues for discourse and transparency (Pasquale 2015) and would allow organizations to critically evaluate the underlying assumptions and implications of algorithm-based HR decision-making tools from a different perspective (Ananny and Crawford 2018; Angwin 2016). However, Manders-Huits and Zimmer (2009) have suggested that to successfully implement a participatory design process, one needs to explicitly address the involved actors’ competing values, engage advocates of those values, and deliberately justify a hierarchy of those values for any given design choice. Furthermore, it might be useful to consider the intricate balance between compliance and integrity when creating such a participatory design process. The integrity perspective would suggest that factors, such as employees’ autonomy, respect, and fairness are important, while the compliance approach would hint toward more formal aspects, such as codes of conduct, along with industry and legal standards to protect employees’ personal integrity (IEEE 2017).

A participatory design approach could avoid many of the pitfalls described in our manuscript. The ideal starting point of any meaningful employee participation is the conceptual stage, before HR and IT have made a purchasing decision. Instead of leaving this decision to technical experts within the organization, HR management should inform employees of its plans, and then consider the values voiced in the process. At the empirical stage, HR management could investigate how employees prioritize certain values, such as privacy and autonomy, when they interact with monitoring systems. In doing so, organizations could also consult with employees regarding the question of what indicators they think should be measured, and why. These values could then be adapted into the software design. Currently, however, most algorithm-based HR decision-making tools available on the market take a “one size fits all” approach, and it is up to consultants and technical experts within organizations to implement a customized version of such a product. In the process of customization lies another opportunity for employee participation. However, this customization must be enabled post-production at the customer site. As detailed above, this requires HR departments to be technologically literate and willing to cooperate with IT departments. Those within the IT department must also, in turn, be ethically aware so that they can identify the potential ethical pitfalls of the algorithms. If this can be accomplished, value-sensitive design processes can generate organizational learning outcomes that benefit all stakeholders involved. If taken seriously, this process can ultimately bolster trust and personal integrity against challenges posed by algorithm-based HR decision-making.

Finally, private regulatory regimes might help in designing and applying algorithm-based HR decision-making tools in more ethically sensitive ways. The use of algorithm-based HR decision-making is a complex challenge that cannot be tackled solely at the level of single corporate actors. Thus, economic actors within civil society, such as corporations, unions, and associations, are called upon to broaden government standards via private regulatory regimes (Wood and Logsdon 2008). The recent update of the Association of Computing Machinery’s (ACM) code of ethics and professional conduct can serve as a good example, as this code already addresses issues such as “fundamental human rights” and “individual’s right to autonomy” (ACM 2018). Another example of such private regulatory regimes is the Toronto Declaration of Machine Learning (Amnesty International and Access Now 2018) which also address such ethical concerns. If corporate actors internalize and act upon these principles (for example in recruitment, employee development, or performance appraisals) this may influence, how algorithm-based HR decision-making tools are developed in the long run. In this manner, regulatory regimes can help generate a better understanding for the involved actors of how algorithm-based HR decisions-making can be both efficient, and at the same time ethically sound.

## Conclusion

Algorithm-based HR decision-making can help monitor employees more effectively but can, at the same time, be ethically problematic. Illustrating the current state of algorithm-based decision-making in HR, we described potential biases and the cultural background of such decision-making tools that can manifest an illusion of control. As a consequence, we suggested that algorithm-based HR decision-making may harm employees’ personal integrity because it can evoke blind trust in processes and rules, which may ultimately marginalize human sense-making as part of their own decision-making processes. This is particularly true because algorithms lack the capacity for moral imagination. To cushion the challenges related to algorithm-based HR decision-making, we emphasized the importance of critical data literacy and ethical awareness and recommended the use of participatory design methods and regulatory regimes.

## References

[CR1] ACM. (2018). ACM code of ethics and professional conduct. https://www.acm.org/code-of-ethics. Accessed September 23, 2018.

[CR2] Adler PS, Borys B (1996). Two types of bureaucracy: Enabling and coercive. Administrative Science Quarterly.

[CR3] AI4ALL. (2018). AI will change the world. Who will change AI? http://ai-4-all.org/. Accessed April 9, 2018.

[CR4] Ajunwa I, Crawford K, Schultz J (2017). Limitless worker surveillance. California Law Review.

[CR5] Alder GS, Ambrose ML (2005). An examination of the effect of computerized performance monitoring feedback on monitoring fairness, performance, and satisfaction. Organizational Behavior and Human Decision Processes.

[CR6] Amnesty International and Access. (2018). The Toronto declaration: Protecting the right to equality and non-discrimination in machine learning systems. https://www.amnesty.org/download/Documents/POL3084472018ENGLISH.PDF.

[CR7] Amoore L, Piotukh V (2015). Algorithmic life: Calculative devices in the age of big data.

[CR8] Ananny M (2016). Toward an ethics of algorithms: Convening, observation, probability, and timeliness. Science, Technology and Human Values.

[CR9] Ananny M, Crawford K (2018). Seeing without knowing: Limitations of the transparency ideal and its application to algorithmic accountability. New Media & Society.

[CR10] Angrave D, Charlwood A, Kirkpatrick I, Lawrence M, Stuart M (2016). HR and analytics: Why HR is set to fail the big data challenge. Human Resource Management Journal.

[CR11] Angwin, J. (2016). Make algorithms accountable. *The New York Times* (Vol. 2018).

[CR12] Ball K (2001). Situating workplace surveillance: Ethics and computer based performance monitoring. Ethics and Information Technology.

[CR13] Ball K (2010). Workplace surveillance: An overview. Labor History.

[CR14] Ball K, Margulis ST (2011). Electronic monitoring and surveillance in call centres: A framework for investigation. New Technology, Work and Employment.

[CR15] Barbrook R, Cameron A (1996). The californian ideology. Science as Culture.

[CR16] Barocas S, Selbst AD (2016). Big data’s disparate impact. California Law Review.

[CR17] Bauman DC (2013). Leadership and the three faces of integrity. The Leadership Quarterly.

[CR18] Becker TE (1998). Integrity in organizations: Beyond honesty and conscientiousness. Academy of Management Review.

[CR19] Beer D (2017). The social power of algorithms. Information, Communication & Society.

[CR20] Bernstein ES (2017). Making transparency transparent: The evolution of observation in management theory. Academy of Management Annals.

[CR21] Beschorner T (2006). Ethical theory and business practices: The case of discourse ethics. Journal of Business Ethics.

[CR22] Bhattacharya S, Wang Y, Xu D (2010). Beyond simon’s means-ends analysis: Natural creativity and the unanswered ‘why’ in the design of intelligent systems for problem-solving. Minds and Machines.

[CR23] Bhimani A, Willcocks L (2014). Digitisation, ‘big data’ and the transformation of accounting information. Accounting and Business Research.

[CR24] Bilić P (2016). Search algorithms, hidden labour and information control. Big Data & Society.

[CR25] Boström N (2014). Superintelligence: Paths, dangers, strategies.

[CR26] boyd d, Crawford K (2012). Critical questions for big data: Provocations for a cultural, technological, and scholarly phenomenon. Information, Communication & Society.

[CR27] Bucher T (2012). Want to be on the top? Algorithmic power and the threat of invisibility on facebook. New Media & Society.

[CR28] Bucher T (2017). The algorithmic imaginary: Exploring the ordinary affects of facebook algorithms. Information, Communication & Society.

[CR29] Buluswar, M., Campisi, V., Gupta, A., Karu, Z., Nilson, V., & Sigala, R. (2016). How companies are using big data and analytics. https://www.mckinsey.com/business-functions/mckinsey-analytics/our-insights/how-companies-are-using-big-data-and-analytics. Accessed September 23, 2018.

[CR30] Buolamwini, J., & Gebru, T. (2018). Gender shades: Intersectional accuracy disparities in commercial gender classification. In *Conference on fairness, accountability and transparency, 2018* (pp. 77–91).

[CR31] Burrell J (2016). How the machine ‘thinks’: Understanding opacity in machine learning algorithms. Big Data & Society.

[CR157] Busch T, Shepherd T (2014). Doing well by doing good? Normative tensions underlying Twitter’s corporate social responsibility ethos. Convergence: The International Journal of Research into New Media Technologies.

[CR32] Calhoun C (1995). Standing for something. The Journal of Philosophy.

[CR33] Carah N (2015). Algorithmic brands: A decade of brand experiments with mobile and social media. New Media & Society.

[CR34] Constantiou ID, Kallinikos J (2015). New games, new rules: Big data and the changing context of strategy. Journal of Information Technology.

[CR35] Crawford, K. (2013). The hidden biases in big data. *Havard Business Review* (Vol. 2018).

[CR36] Crawford K (2015). Can an algorithm be agonistic? Ten scenes from life in calculated publics. Science, Technology and Human Values.

[CR37] Crawford, K. (2016). Artificial intelligence’s white guy problem. *The New York Times* (Vol. 2018).

[CR38] Davenport TH (2013). Analytics 3.0. Harvard Business Review.

[CR39] Delle Donne B (2017). Guiding talent acquisition technology into the future 2017 State of Talent Acquisition Technology.

[CR40] Devlin, H. T. G. (2017). AI programs exhibit racial and gender biases, research reveals. https://www.theguardian.com/technology/2017/apr/13/ai-programs-exhibit-racist-and-sexist-biases-research-reveals?CMP=twt_gu. Accessed September 23, 2018.

[CR41] Diakopoulos N (2016). Accountability in algorithmic decision making. Communications of the ACM.

[CR42] Dourish P (2016). Algorithms and their others: Algorithmic culture in context. Big Data & Society.

[CR43] Dovey J, Kennedy HW (2006). Game cultures: Computer games as new media: Computer games as new media.

[CR44] Durand R (2003). Predicting a firm’s forecasting ability: The roles of organizational illusion of control and organizational attention. Strategic Management Journal.

[CR45] Ekbia H, Mattioli M, Kouper I, Arave G, Ghazinejad A, Bowman T (2015). Big data, bigger dilemmas: A critical review. Journal of the Association for Information Science and Technology.

[CR46] Forster N, Cebis M, Majteles S, Mathur A, Morgan R, Preuss J (1999). The role of story-telling in organizational leadership. Leadership & Organization Development Journal.

[CR47] Foucault M (1977). Discipline and punish: The birth of the prison.

[CR48] Fox S (1989). The panopticon: From Bentham’s obsession to the revolution in management learning. Human Relations.

[CR49] Friedman B, Kahn PH, Borning A, Huldtgren A, Zhang P, Galletta D (2013). Value sensitive design and information systems. Early engagement and new technologies: Opening up the laboratory.

[CR50] Galič M, Timan T, Koops B-J (2017). Bentham, deleuze and beyond: An overview of surveillance theories from the panopticon to participation. Philosophy & Technology.

[CR51] Gardner WL, Fischer D, Hunt JG (2009). Emotional labor and leadership: A threat to authenticity?. The Leadership Quarterly.

[CR52] Garson B (1989). Electronic sweatshop: How computers are transforming the office of the future into the factory of the past.

[CR53] Gillespie T, Gillespie T, Boczkowski P, Foot KA (2014). The relevance of algorithms. Media technologies: Essays on communication, materiality, and society.

[CR54] Goodstein JD (2000). Moral compromise and personal integrity: Exploring the ethical issues of deciding together in organizations. Business Ethics Quarterly.

[CR55] Google. (2017). Machine learning and human bias. youtube.com.

[CR56] Greenwood MR (2002). Ethics and HRM: A review and conceptual analysis. Journal of Business Ethics.

[CR57] Gutmann A, Thompson D (1996). Democracy and disagreement.

[CR58] Hallinan B, Striphas T (2014). Recommended for you: The netflix prize and the production of algorithmic culture. New Media & Society.

[CR59] Hasselbalch G, Tranberg P (2016). Data ethics: The new competitive advantage.

[CR60] IBM. (2018). Power your candidate experience with AI. https://twitter.com/IBMWatsonTalent?lang=en. Accessed September 23, 2018.

[CR61] IEEE (2017). Prioritizing human well-being in the age of artificial intelligence.

[CR62] Introna LD (2015). Algorithms, governance, and governmentality: On governing academic writing. Science, Technology and Human Values.

[CR63] Jasanoff S (2016). The ethics of invention: Technology and the human future.

[CR156] Johnson DG (1994). Computer ethics.

[CR64] Kitchin R (2017). Thinking critically about and researching algorithms. Information, Communication & Society.

[CR65] Koehn D (2005). Integrity as a business asset. Journal of Business Ethics.

[CR66] Konrad, A. (2013). Meet orion, software that will save ups millions by improving drivers’ routes. *Forbes* (Vol. 2018).

[CR67] Kottemann JE, Davis FD, Remus WE (1994). Computer-assisted decision making: Performance, beliefs, and the illusion of control. Organizational Behavior and Human Decision Processes.

[CR68] KPMG. (2015). Evidence-based HR: The bridge between your people and delivering business strategy. https://assets.kpmg.com/content/dam/kpmg/pdf/2015/04/evidence-based-hr.pdf. Accessed September 23, 2018.

[CR69] Krumeich J, Werth D, Loos P (2016). Prescriptive control of business processes. Business & Information Systems Engineering.

[CR70] Kushner S (2013). The freelance translation machine: Algorithmic culture and the invisible industry. New Media & Society.

[CR71] Leclercq-Vandelannoitte AL (2017). An ethical perspective on emerging forms of ubiquitous it-based control. Journal of Business Ethics.

[CR72] Lee MK (2018). Understanding perception of algorithmic decisions: Fairness, trust, and emotion in response to algorithmic management. Big Data & Society.

[CR73] Legge K (1996). Morality bound. People Management.

[CR74] Lowrie I (2017). Algorithmic rationality: Epistemology and efficiency in the data sciences. Big Data & Society.

[CR75] Lycett M (2013). ‘Datafication’: Making sense of (big) data in a complex world. European Journal of Information Systems.

[CR76] Mager A (2012). Algorithmic ideology. Information, Communication & Society.

[CR77] Manders-Huits N, Zimmer M (2009). Values and pragmatic action: The challenges of introducing ethical intelligence in technical design communities. International Review of Information Ethics.

[CR78] Margolis JD, Grant AM, Molinsky AL (2007). Expanding ethical standards of HRM: Necessary evils and the multiple dimensions of impact.

[CR79] Martin K (2016). Understanding privacy online: Development of a social contract approach to privacy. Journal of Business Ethics.

[CR80] Martin K (2018). Ethical implications and accountability of algorithms. Journal of Business Ethics.

[CR81] Martin K, Freeman RE (2003). Some problems with employee monitoring. Journal of Business Ethics.

[CR82] Martin K, Freeman RE (2004). The separation of technology and ethics in business ethics. Journal of Business Ethics.

[CR83] Martin K, Nissenbaum H (2016). Measuring privacy: An empirical test using context to expose confounding variables. Columbia Science and Technology Law Review.

[CR84] McFall L (1987). Integrity. Ethics.

[CR85] Miller P (1996). Strategy and the ethical management of human resources. Human Resource Management Journal.

[CR86] Mittelstadt BD, Allo P, Taddeo M, Wachter S, Floridi L (2016). The ethics of algorithms: Mapping the debate. Big Data & Society.

[CR87] Möhlmann, M., & Zalmanson, L. (2017). Hands on the wheel: Navigating algorithmic management and uber drivers’ autonomy. In *International conference on information systems (ICIS 2017), Seoul, South Korea*, December 10–13, 2017.

[CR88] Morozov, E. (2013). The perils of perfection. *The New York Times* (Vol. 2018).

[CR89] Neyland D (2015). Bearing account-able witness to the ethical algorithmic system. Science, Technology and Human Values.

[CR90] Neyland D, Möllers N (2017). Algorithmic if … then rules and the conditions and consequences of power. Information, Communication & Society.

[CR91] Nijhuis, M. (2017). How to call B.S. on big data: A practical guide *The New Yorker* (Vol. 2018).

[CR92] Noble SU (2018). Algorithms of oppression: How search engines reinforce racism.

[CR93] O’Heigeartaigh, S. (2013). Would you hand over a moral decision to a machine? Why not? Moral outsourcing and artificial intelligence. In *Practical ethics*. Oxford: University of Oxford.

[CR94] O’Neil C (2016). Weapons of math destruction: How big data increases inequality and threatens democracy.

[CR95] Orlikowski WJ (2007). Sociomaterial practices: Exploring technology at work. Organization Studies.

[CR96] Ottensmeyer EJ, Heroux MA (1991). Ethics, public policy, and managing advanced technologies: The case of electronic surveillance. Journal of Business Ethics.

[CR97] Paine LS (1994). Managing for organizational integrity. Harvard Business Review.

[CR98] Palanski ME, Yammarino FJ (2009). Integrity and leadership: A multi-level conceptual framework. The Leadership Quarterly.

[CR99] Parry KW, Cohen M, Bhattacharya S (2016). Rise of the machines: A critical consideration of automated leadership decision making in organizations. Group & Organization Management.

[CR100] Parry KW, Hansen H (2007). The organizational story as leadership. Leadership.

[CR101] Parry KW, Proctor-Thomson SB (2002). Perceived integrity of transformational leaders in organisational settings. Journal of Business Ethics.

[CR102] Pasquale F (2015). The black box society: The secret algorithms that control money and information.

[CR103] Peck D (2013). They’re watching you at work. The Atlantic.

[CR104] Porter TM (1996). Trust in numbers. The pursuit of objectivity in science and public life.

[CR105] Rasche A, Esser D (2006). From stakeholder management to stakeholder accountability. Journal of Business Ethics.

[CR106] Rasche A, Esser DE, Carter C, Clegg S, Kornberger M, Laske S, Messner M (2007). Managing for compliance and integrity in practice. Business ethics as practice. Representation, reflexivity and performance.

[CR107] Rieke ML, Guastello SJ (1995). Unresolved issues in honesty and integrity testing. American Psychologist.

[CR108] Rosenblat, A., Kneese, T., & boyd, d. (2014). *Workplace surveillance*. Data & Society Working Paper. New York: Data & Society Research Institute.

[CR109] Rosenblat A, Stark L (2016). Algorithmic labor and information asymmetries: A case study of uber’s drivers. International Journal of Communication.

[CR110] Rule JB, Lyon D, Zureik E (1996). High-tech workplace surveillance: What’s really new?. Computers, surveillance, and privacy.

[CR111] Ryan RM, Deci EL (2000). Self-determination theory and the facilitation of intrinsic motivation, social development, and well-being. American Psychologist.

[CR112] SAS. (2018). Becoming a data-driven organization. https://www.sas.com/sas/offers/17/becoming-a-data-driven-organization-2582640.html?gclid=Cj0KCQjwqM3VBRCwARIsAKcekb1_bbm111yzeM-F1hr1F31dpslA9do0sNqUC72VZaeigFNGJTbXaY0aApFKEALw_wcB. Accessed September 23, 2018.

[CR113] Saval N (2014). Cubed: A secret history of the workplace.

[CR114] Scherer AG (2015). Can hypernorms be justified? Insights from a discourse—ethical perspective. Business Ethics Quarterly.

[CR115] Seaver N (2017). Algorithms as culture: Some tactics for the ethnography of algorithmic systems. Big Data & Society.

[CR116] Sharma R, Mithas S, Kankanhalli A (2014). Transforming decision-making processes: A research agenda for understanding the impact of business analytics on organisations. European Journal of Information Systems.

[CR117] Shilton K, Anderson S (2017). Blended, not bossy: Ethics roles, responsibilities and expertise in design. Interacting with Computers.

[CR118] Simonite, T. (2012). Microsoft’s workplace social network becomes emotionally aware. *MIT Technology Review* (Vol. 2018).

[CR119] Simons T (2002). Behavioral integrity: The perceived alignment between managers’ words and deeds as a research focus. Organization Science.

[CR120] Son, H. (2015). JPMorgan algorithm knows you’re a rogue employee before you do. http://www.bloomberg.com/news/articles/2015-04-08/jpmorgan-algorithm-knows-you-re-a-rogue-employee-before-you-do. Accessed June 15, 2016.

[CR121] Souza GC (2014). Supply chain analytics. Business Horizons.

[CR122] Stanton JM (2000). Reactions to employee performance monitoring: Framework, review, and research directions. Human Performance.

[CR123] Stewart TR, McMillan C, Stewart TR, McMillan C (1987). Descriptive and prescriptive models for judgment and decision making: Implications for knowledge engineering. Expert judgment and expert systems.

[CR124] Stohl C, Stohl M, Leonardi PM (2016). Digital age|managing opacity: Information visibility and the paradox of transparency in the digital age. International Journal of Communication.

[CR125] Striphas T (2015). Algorithmic culture. European Journal of Cultural Studies.

[CR126] Tarnoff, B. (2017). Silicon Valley siphons our data like oil. But the deepest drilling has just begun. *The Guardian* (Vol. 2018).

[CR127] Taylor C (2007). A secular age.

[CR155] Taylor JR, Van Every EJ (2000). The emergent organization: Communication as its site and surface.

[CR128] Thelwall M (2018). Gender bias in machine learning for sentiment analysis. Online Information Review.

[CR129] Thomas SL, Nafus D, Sherman J (2018). Algorithms as fetish: Faith and possibility in algorithmic work. Big Data & Society.

[CR130] Thorp, J. (2012). Big data is not the new oil. *Harvard Business Review* (Vol. 2018).

[CR131] Tomlinson EC, Lewicki RJ, Ash SR (2014). Disentangling the moral integrity construct: Values congruence as a moderator of the behavioral integrity–citizenship relationship. Group & Organization Management.

[CR132] Turkle S (1995). Life on the screen: Identity in the age of the internet.

[CR133] Van der Velden M, Mörtberg C, Van den Hoven J, Vermaas PE, Van de Poel I (2015). Participatory design participatory and design for values. Handbook of ethics, values, and technological design.

[CR134] Van Dijck J (2014). Datafication, dataism and dataveillance: Big data between scientific paradigm and ideology. Surveillance & Society.

[CR135] Vara, V. (2015). Can Intel make silicon valley more diverse. *The New Yorker* (Vol. 2018).

[CR136] Verbeek P-P (2006). Materializing morality: Design ethics and technological mediation. Science, Technology and Human Values.

[CR137] Verhezen P (2010). Giving voice in a culture of silence. From a culture of compliance to a culture of integrity. Journal of Business Ethics.

[CR138] Vidaver-Cohen D (1997). Moral imagination in organizational problem-solving: An institutional perspective. Business Ethics Quarterly.

[CR139] Watson, R. (2016). In silicon valley, young white males are stealing the future from everyone else. *The Guardian* (Vol. 2018).

[CR140] Weibel A (2007). Formal control and trustworthiness: Shall the twain never meet?. Group & Organization Management.

[CR141] Weibel A, Six F, Bachmann R, Zaheer A (2013). Trust and control: The role of intrinsic motivation. Handbook of advances in trust.

[CR142] Weibel A, Wildhaber I, Leicht-Deobald U, Schank C, Busch T, Gallen UOS (2016). Big data or big brother?—Big data hr control practices and employees’ trust in the employer. Unpublished grant proposal.

[CR143] Weick KE, Sutcliffe KM, Obstfeld D (2005). Organizing and the process of sensemaking. Organization Science.

[CR144] Werhane PH (1998). Moral imagination and the search for ethical decision-making in management. Business Ethics Quarterly.

[CR145] Werhane PH (1999). Moral imagination and management decision making.

[CR146] Why, M. (2018). *4 reasons why an automated hiring process will help your company*. Select International, a psi business (Vol. 2018). Pittsburg, PA: Select International.

[CR147] Wilcox T (2012). Human resource management in a compartmentalized world: Whither moral agency?. Journal of Business Ethics.

[CR148] Willson M (2017). Algorithms (and the) everyday. Information, Communication & Society.

[CR149] Wood DJ, Logsdon JM (2008). Business citizenship as metaphor and reality. Business Ethics Quarterly.

[CR150] Zarsky T (2015). The trouble with algorithmic decisions: An analytic road map to examine efficiency and fairness in automated and opaque decision making. Science, Technology and Human Values.

[CR151] Zax, D. (2013). Brown down: UPS drivers vs. The UPS algorithm. *FastCompany* (Vol. 2019).

[CR152] Ziewitz M (2015). Governing algorithms: Myth, mess, and methods. Science, Technology and Human Values.

[CR153] Zuboff S (1988). In the age of the smart machine: The future of work and power.

[CR154] Zuboff S (2015). Big other: Surveillance capitalism and the prospects of an information civilization. Journal of Information Technology.

